# Identification of *RhoGAP* Gene Family in Soybean (*Glycine max* L.) and Its Role in the Response to *Rhizobium* Infection

**DOI:** 10.3390/ijms27146239

**Published:** 2026-07-13

**Authors:** Chengcheng Qin, Han Huang, Yanbo Sun, Ruixue Luo, Xin Zhang, Bohong Su, Jian Song

**Affiliations:** 1 College of Life Sciences, Yangtze University, Jingzhou 434025, China; 2 College of Agronomy and Biotechnology, China Agricultural University, Beijing 100193, China; 3 MARA Key Laboratory of Sustainable Crop Production in the Middle Reaches of the Yangtze River (Co-Construction by Ministry and Province)/Hubei Key Laboratory of Waterlogging Disaster and Agricultural Use of Wetland, College of Agriculture, Yangtze University, Jingzhou 434025, China

**Keywords:** *RhoGAP*, soybean, rhizobial infection, gene expression analysis

## Abstract

Rho GTPase-activating proteins (RhoGAPs) are characterized by a conserved RhoGAP domain and function as negative regulators of Rho GTPases, playing important roles in plant growth, development, and responses to environmental stimuli. In this study, 19 *GmRhoGAP* genes were identified in the soybean genome and found to be unevenly distributed across 11 chromosomes. Comprehensive analyses were performed, including gene structure, conserved motifs, protein domains, gene duplication, synteny, *cis*-acting elements, tissue-specific expression, and quantitative real-time PCR under rhizobial infection. Structural analysis revealed substantial diversity in intron-exon organization but high conservation of motif composition, with all members containing the conserved RhoGAP domain. A total of 20 segmentally duplicated gene pairs were identified, indicating expansion of the *GmRhoGAP* family in soybean. Inter-species synteny analysis showed closer evolutionary relationships with *Arabidopsis* than with rice, and Ka/Ks analysis suggested strong purifying selection during evolution. Promoter analysis indicated potential involvement in development, phytohormone signaling, stress responses, and light responsiveness. Expression profiling demonstrated distinct tissue-specific patterns. qRT-PCR further showed that *GmRhoGAP* genes respond differentially to rhizobial infection, with 10 generally upregulated genes and 4 downregulated genes. These analyses provide a framework for understanding this gene family and identify candidate genes for future research.

## 1. Introduction

Rho GTPases are a class of small G proteins of approximately 21 kDa belonging to the Ras superfamily [1]. These proteins function as molecular switches by cycling between an inactive GDP-bound state and an active GTP-bound state, thereby regulating fundamental biological processes such as cell polarity establishment, cell morphogenesis, and cytoskeletal reorganization [2,3]. In animals, the Rho family is typically divided into three major subfamilies—Rho, Rac, and Cdc42; plants possess a single Rho-related small GTPase family, namely ROPs (Rho-like GTPases from Plants). ROPs are sometimes also referred to as RACs, as their amino acid sequences are most homologous to those of animal RAC proteins [4]. The GDP/GTP cycling of Rho GTPases is tightly regulated by the coordinated actions of guanine nucleotide exchange factors (GEFs), GTPase-activating proteins (GAPs), and guanine nucleotide dissociation inhibitors (GDIs) [5]. Additionally, plants harbor a set of specialized regulators, including PRONE-type GEFs (termed RopGEFs) and effectors such as RICs and ICRs/RIPs, which function in regulating the cytoskeleton and vesicle trafficking [4]. Among them, GAPs promote the hydrolysis of GTP to inactivate Rho GTPases, playing a critical role in the regulation of their activity [6].

Rho GTPase-activating proteins (RhoGAPs) family contain a conserved GAP catalytic domain, which consists of approximately 150 amino acid residues that typically fold into 7 α-helices [7]. During catalysis, GAPs provide an “arginine finger” that stabilizes the partial negative charge developed in the transition state of GTP hydrolysis, while simultaneously inducing a conformational change in the conserved Gln63 residue of the Rho protein and activating a water molecule to perform an in-line nucleophilic attack on the γ-phosphate of GTP, thereby forming a highly efficient catalytic site [8,9].

To date, two types of RhoGAP proteins have been identified in plants: RopGAPs and REN (also referred to as PHGAPs). Although both types contain a conserved RhoGAP domain, they differ significantly in structural organization and sequence features [10,11]. RopGAPs possess an N-terminal Cdc42/Rac-interactive binding (CRIB) motif. In plants, this domain is specifically integrated into RopGAPs to enhance their recognition and binding affinity for activated Rop GTPases, and is involved in the subcellular localization of RopGAPs; in animals, the CRIB domain is typically found in Cdc42/Rac effector proteins [4,5]. REN proteins contain an N-terminal pleckstrin homology (PH) domain and two coiled-coil domains at their C-terminus [10,12]. The PH domain, which consists of 100–120 amino acids, interacts with phosphoinositides but does not participate in the subcellular localization of REN [10]. Previous studies have shown that REN1, a member of the PHGAP family, acts as a global inhibitor that spatially restricts ROP1 activity to the apical plasma membrane (PM) [10]. To date, six RopGAPs and three PHGAPs have been identified in *Arabidopsis thaliana* (*A. thaliana*) [5]. In plants, RhoGAPs are involved in fundamental biological processes such as cell polarity establishment, polar growth, and morphogenesis, as well as in environmental responses, through the precise spatiotemporal regulation of Rop GTPase activity. Previous studies indicate that RhoGAPs play critical roles in polarized cell growth [13,14]. By restricting the spatial activity of Rop proteins, RhoGAPs participate in the regulation of tip-focused intracellular Ca^2+^ gradients, Ca^2+^ influx, and dynamic reorganization of the actin cytoskeleton, thereby maintaining cell polarity [12].

*Glycine max* (*G. max*) has undergone multiple whole-genome duplication (WGDs) events during its evolutionary history, which have facilitated the expansion and functional diversification of numerous gene families. However, a systematic genome-wide identification and evolutionary analysis of the *RhoGAP* gene family in soybean are still lacking. In addition, although previous studies have demonstrated that Rop GTPases participate in symbiotic nitrogen fixation by regulating root hair polarized growth, root hair deformation, rhizobial infection, and nodulation [15,16,17,18], the expression patterns of soybean *RhoGAP* genes during rhizobial infection remain unclear. Therefore, a systematic identification and comprehensive characterization of the soybean *RhoGAP* gene family, including its phylogenetic relationships, gene structures, chromosomal distribution, duplication events, *cis*-regulatory elements, and expression patterns in response to rhizobial infection, will not only help elucidate the expansion and divergence mechanisms of this family in legumes, but also provide valuable gene resources for future functional studies on legume–*Rhizobium* symbiosis.

## 2. Results

### 2.1. Identification of RhoGAP Genes in the Soybean Genome

In this study, 19 *GmRhoGAP* genes were identified in the soybean genome using the amino acid sequence of RhoGAP1 from *Oryza sativa* (*O. sativa*) as a reference template. The *GmRhoGAP* family members were named *GmRhoGAP1* to *GmRhoGAP19* based on their chromosomal positions (Table S3). Relevant information for these *GmRhoGAP* genes and their corresponding proteins is listed in Table S4. The number of amino acid (aa) ranged from 151 to 946 aa; molecular weights (MW) varied from 16.81 kDa to 103.54 kDa; theoretical isoelectric points (pI) ranged from 4.38 to 8.30; instability indices (II) ranged from 48.00 to 65.15; aliphatic indices (AI) ranged from 70.35 to 106.62; and grand average of hydropathicity (GRAVY) values ranged from −0.83 to 0.21.

Overall, these 19 GmRhoGAP proteins exhibited significant variation in length, charge, stability, and hydrophobicity, with most being acidic, hydrophilic, and unstable proteins. Notably, GmRhoGAP12 exhibited the shortest amino acid sequence, the smallest molecular weight, the highest aliphatic index, and was the only hydrophobic protein in the family.

### 2.2. Phylogenetic Analysis of the GmRhoGAP Gene Family

**Figure 1:**
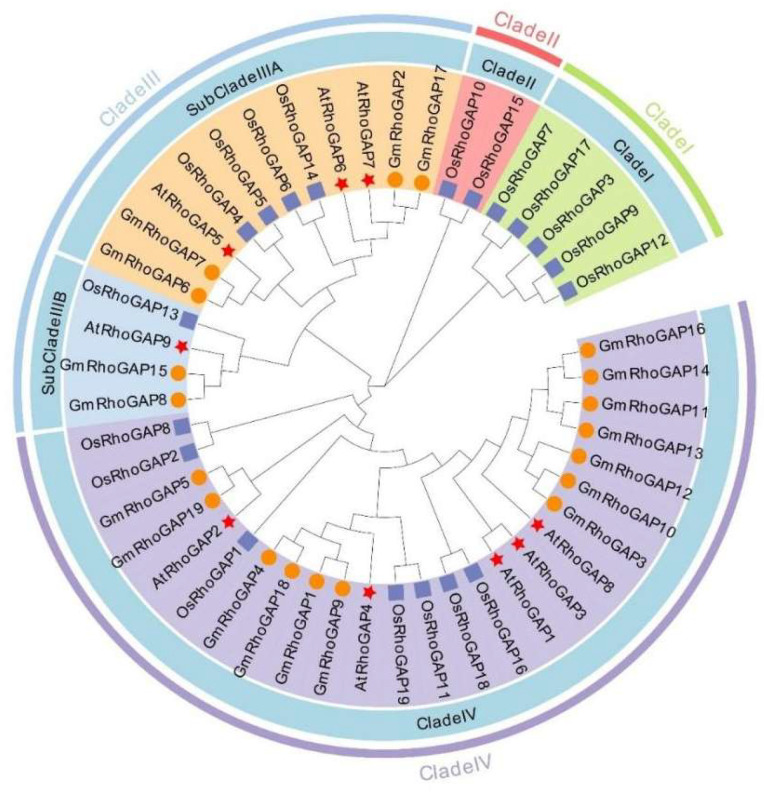
Phylogenetic tree of RhoGAP protein sequences from *A. thaliana*, *O. sativa*, and *G. max*. The phylogenetic tree of RhoGAP proteins from *A. thaliana* (At; red pentagrams), *G. max* (Gm; yellow circles), and *O. sativa* (Os; blue squares) was constructed using the Neighbor-Joining (NJ) method in MEGA 11, with 1000 bootstrap replicates. Clades I–IV are color-coded for distinction.

To explore the phylogenetic relationships of the RhoGAP family in *A. thaliana*, *O. sativa*, and *G. max*, a phylogenetic tree was constructed using 47 RhoGAP protein sequences from these species (Figure 1). The results revealed that the 47 RhoGAP proteins could be grouped into four clades: Clades I to IV. Additionally, Clade III was further subdivided into two subclades: Clade IIIA and Clade IIIB. Clade IV was the largest clade in the phylogenetic tree, containing 25 members, including 13 GmRhoGAPs, 7 OsRhoGAPs, and 5 AtRhoGAPs. Clade III was the second largest clade, consisting of 6 GmRhoGAPs, 5 OsRhoGAPs, and 4 AtRhoGAPs. Clade I and Clade II each contained only *O. sativa* RhoGAPs, with 5 OsRhoGAPs in Clade I and 2 OsRhoGAPs in Clade II (Figure 1).

### 2.3. Motif Composition, Conserved Domain, and Gene Structure Prediction of GmRhoGAPs

**Figure 2:**
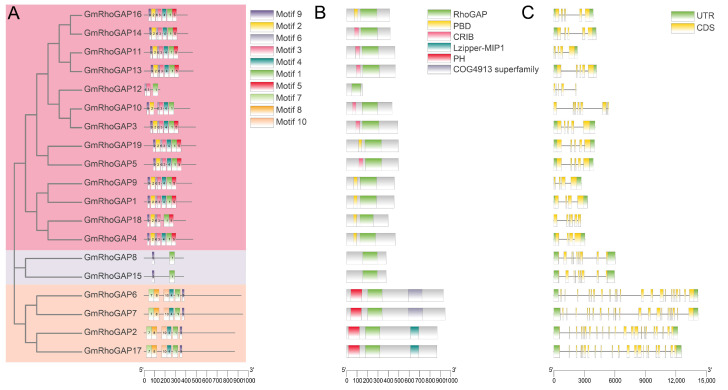
Analysis of conserved motifs, protein domains, and gene structures of GmRhoGAPs. (**A**) Motif composition of GmRhoGAP proteins. Different motifs, numbered 1 to 10, are represented by colored boxes, with protein length estimated using the scale bar at the bottom. (**B**) Conserved domains and their distribution in GmRhoGAP proteins. The names of the conserved domains are labeled below, and each domain is represented by a distinct color. (**C**) Gene structure of *GmRhoGAP* genes. UTRs, coding sequences (CDS), and introns are represented by green boxes, yellow boxes, and gray lines, respectively.

To investigate the structural characteristics and potential functional diversification of the soybean *RhoGAP* gene family, we performed a comprehensive analysis of the phylogenetic relationships, motif composition, conserved domains, and gene structures of the 19 GmRhoGAPs based on coding sequences and genomic information. Phylogenetic and motif analyses revealed that the 19 GmRhoGAPs could be clearly divided into three major clades, with members of the same clade exhibiting highly conserved motif compositions and arrangement patterns. In contrast, members of different clades exhibited significant differences in motif types and their arrangements (Figure 2A and Figure S1). Most of the GmRhoGAPs contained motifs 1, 2, 3, 4, 5, 6, and 9. Conserved domain analysis indicated that all GmRhoGAPs contained a typical RhoGAP domain. In addition to GmRhoGAP8, GmRhoGAP12, and GmRhoGAP15, the remaining proteins contained 1~2 other domains and carried 6 or more motifs (Figure 2A,B).

Gene structure analysis revealed significant differences in the exon–intron organization of *GmRhoGAP* genes. Most members contained 4~9 exons, whereas *GmRhoGAP2*, *GmRhoGAP6*, *GmRhoGAP7*, and *GmRhoGAP17* contained 22~23 exons. Furthermore, with the exception of *GmRhoGAP10*, *GmRhoGAP12*, and *GmRhoGAP18*, which lacked untranslated region (UTR) regions, all other members contained both 5′ and 3′ UTRs (Figure 2C). The *GmRhoGAP* gene family exhibited “high conservation within clades and significant divergence between clades” in terms of phylogenetic grouping, conserved motif composition, domain configuration, and gene structure. These structural differences may contribute to the functional diversification of the *GmRhoGAP* gene family, while the conserved core RhoGAP domain may ensure the stability of its basic biological functions.

### 2.4. Chromosomal Distribution and Synteny Analysis of GmRhoGAPs

**Figure 3:**
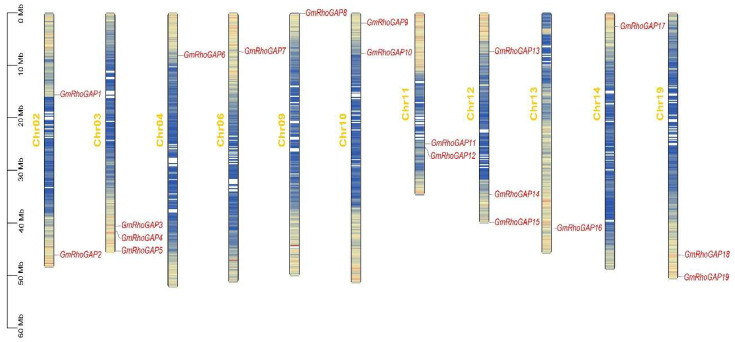
Chromosomal distribution of *GmRhoGAPs.* The *RhoGAP* genes were localized on the corresponding chromosomes based on their physical positions in the soybean genome. The central part of each chromosomal scaffold structure is labeled with numbers. Chromosome sizes are displayed using a vertical scale, with units in Mb. The color of the chromosomes indicates gene density, with red representing the highest gene density and blue representing the lowest gene density.

To investigate the distribution characteristics of *GmRhoGAP* genes on soybean chromosomes, a chromosomal map was constructed. The results showed that the 19 *GmRhoGAP* genes were distributed unevenly and irregularly across 11 soybean chromosomes, with most genes located near the chromosome ends (Figure 3). Specifically, one *GmRhoGAP* gene was located on chromosomes Chr04, Chr06, Chr09, Chr13, and Chr14; two *GmRhoGAP* genes were located on chromosomes Chr02, Chr10, Chr11, and Chr19; three *GmRhoGAP* genes were located on chromosomes Chr03 and Chr12. No genes were found on chromosomes Chr01, Chr05, Chr07, Chr08, Chr15–18, and Chr20 (Figure 3 and Table S3).

**Figure 4:**
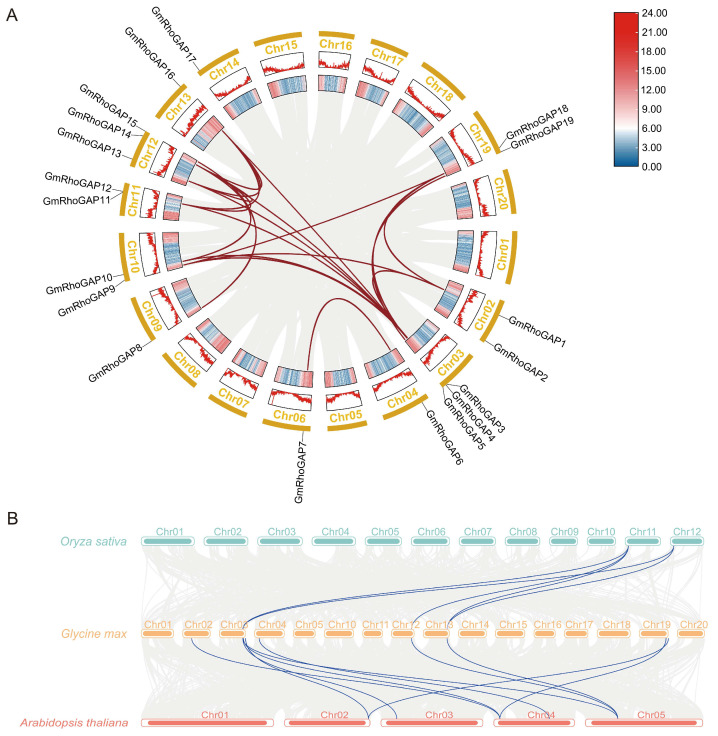
Synteny analysis of the *RhoGAP* gene family in *O. sativa*, *G. max*, and *A. thaliana*. (**A**) Diagram showing the interchromosomal relationships of *GmRhoGAP* genes. Red lines represent syntenic blocks within the soybean genome. (**B**) Synteny analysis of *RhoGAP* genes in *O. sativa*, *G. max*, and *A. thaliana*. Gray lines in the background represent syntenic blocks within the *O. sativa*, *G. max*, and *A. thaliana* genomes. Blue lines indicate syntenic *GmRhoGAP* gene pairs.

Gene duplication is considered one of the most important driving forces of genome evolution [19]. Common types of duplication include tandem duplications, segmental duplications, and interspersed duplications. Among these, segmental duplications and tandem duplications are believed to be major contributors to plant gene family expansion [20]. Tandem duplications are characterized by two adjacent genes located on the same chromosome, usually separated by no more than five genes [21]; segmental duplications typically arise from WGD events, followed by chromosomal rearrangements, resulting in syntenic duplicated blocks between different chromosomes [20]. In this study, intra-species synteny analysis revealed 20 pairs of segmentally duplicated genes across 10 chromosomes (Chr02-04, Chr06, Chr09-13, Chr19) (Figure 4A and Table S5). No tandemly duplicated genes were identified on the soybean chromosomes, suggesting that segmental duplications may play a dominant role in the expansion of this gene family. To further explore the evolutionary relationships of the *GmRhoGAP* gene family, synteny analyses were conducted between the soybean genome and the genomes of *O. sativa* and *A. thaliana*. The results showed that 9 *GmRhoGAP* genes exhibited pairwise synteny with genes in the *Arabidopsis* genome, and 3 *GmRhoGAP* genes showed pairwise synteny with genes in the *Oryza* genome (Figure 4B and Table S6). Notably, *GmRhoGAP3*, *GmRhoGAP14*, and *GmRhoGAP16* had syntenic counterparts in both *Oryza* and *Arabidopsis*, suggesting that these genes may have originated from a common ancestor of angiosperms and have maintained a high degree of conservation during long-term evolution.

To investigate whether Darwinian positive selection played a role in the divergence of *GmRhoGAP* genes after duplication and to trace the timing of these duplication events, the Ka/Ks ratios for the 20 pairs of segmentally duplicated *GmRhoGAP* genes were calculated. The Ks values were used to estimate the approximate timing of the duplication events, with all Ka/Ks ratios detailed in Table S5. The results indicated that the segmental duplication events of *GmRhoGAP* genes in the soybean genome likely occurred between 5.99 Mya (million years ago, Ks = 0.073806) and 232.3 Mya (Ks = 2.862367934), with a mean value of approximately 119.15 Mya (Ks = 0.872029). The Ka/Ks ratios for all 20 pairs of segmentally duplicated genes were less than 1 (Table S5), indicating that purifying selection has played a dominant role in the evolution of this gene family.

### 2.5. Cis-Regulatory Element Analysis of GmRhoGAP Promoters

**Figure 5:**
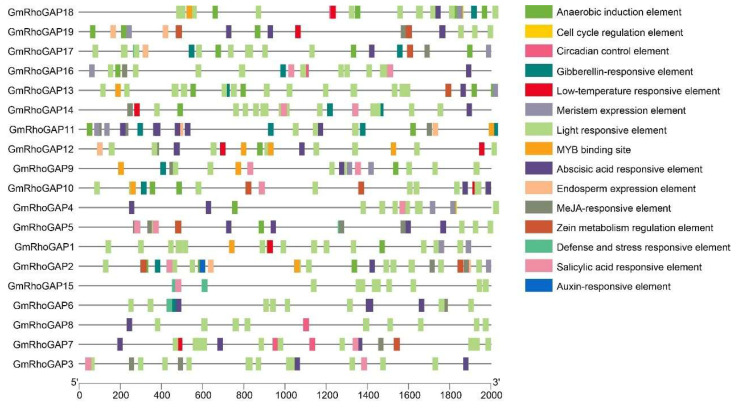
*Cis*-acting element analysis of the promoter regions of *GmRhoGAPs*. The *cis*-elements within the 2000 bp upstream promoter region of each gene were mapped onto the gene sequences. Different types of *cis*-elements are represented by distinct colors as indicated on the right side of the figure. The length of the promoter sequences is indicated by the scale bar at the bottom of the figure.

*Cis*-regulatory elements play a crucial role in the transcriptional regulation of gene expression. In this study, a total of 15 types of *cis*-regulatory elements were identified in the promoter regions of *GmRhoGAPs*. These elements can be classified into four major categories: light-responsive elements, plant hormone-responsive elements, environmental stress-responsive elements, and plant growth- and development-related elements (Figure 5 and Table S7). Notably, a large number of light-responsive elements were found in the promoter regions, suggesting that *GmRhoGAP* genes may be involved in light signaling pathways. Additionally, numerous hormone-related elements were identified, including auxin (IAA)-responsive, salicylic acid (SA)-responsive, gibberellin (GA)-responsive, abscisic acid (ABA)-responsive, and methyl jasmonate (MeJA)-responsive elements (Table S7). Other identified defense-, stress-, anaerobic-, and low-temperature-responsive elements in the promoters indicate that *GmRhoGAPs* may be involved in a variety of biological processes and may exhibit different responses to abiotic stresses. Regarding plant growth and development, elements related to meristem and endosperm expression were identified, suggesting that *GmRhoGAPs* may be associated with seed development and storage material accumulation.

### 2.6. Expression Patterns of GmRhoGAPs in Different Tissues

**Figure 6:**
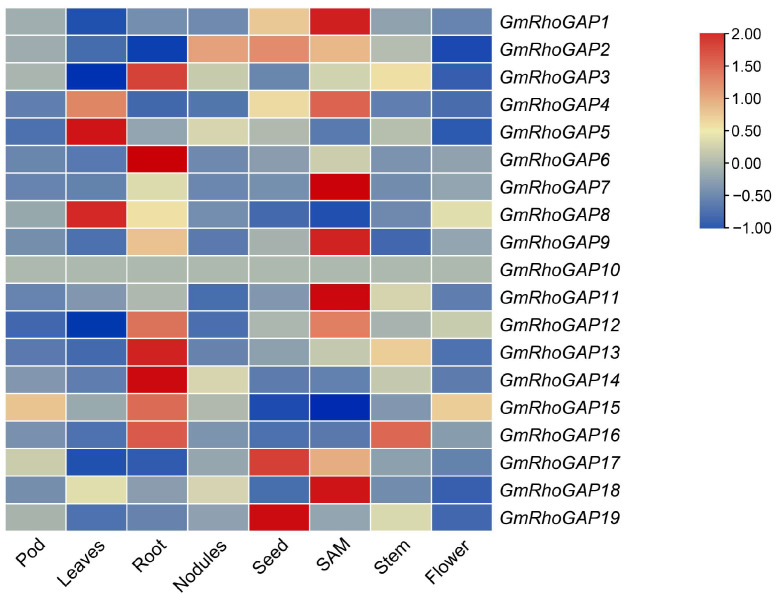
Expression patterns of *GmRhoGAP* genes in different tissues. Expression data were obtained from the Phytozome 14 database (https://phytozome-next.jgi.doe.gov/info/Gmax_Wm82_a4_v1 accessed on 7 March 2026). The color scale from blue to red represents low to high expression levels.

Tissue-specific expression analysis revealed that, except for *GmRhoGAP10*, the expression levels of the remaining *GmRhoGAP* genes varied significantly across different tissues (Figure 6 and Table S8). Based on expression patterns, these genes can be classified into two groups: one group with tissue-specific expression (14 *GmRhoGAPs*) and the other with high expression across multiple tissues (4 *GmRhoGAPs*). Notably, *GmRhoGAP5* and *GmRhoGAP8* showed tissue-specific high expression in leaves; additionally, five *GmRhoGAP* genes exhibited high expression specifically in root and shoot apical meristem (SAM) (*GmRhoGAP3*, *GmRhoGAP6*, *GmRhoGAP13*, *GmRhoGAP14*, and *GmRhoGAP15* in root; *GmRhoGAP1*, *GmRhoGAP7*, *GmRhoGAP9*, *GmRhoGAP11*, and *GmRhoGAP18* in SAM), which together account for more than half of the *GmRhoGAP* gene family. Furthermore, *GmRhoGAP17* and *GmRhoGAP19* exhibited tissue-specific high expression in seed. In contrast, *GmRhoGAP2*, *GmRhoGAP4*, *GmRhoGAP12*, and *GmRhoGAP16* displayed relatively high expression levels across multiple tissues (Figure 6 and Table S8). Specifically, *GmRhoGAP2* maintained high expression levels in nodules, seeds, and SAM; *GmRhoGAP12* showed relatively low expression in pods, leaves, and nodules, but high expression in the remaining tissues. It is noteworthy that *GmRhoGAP4* and *GmRhoGAP16* had significantly higher expression in leaves and SAM (for *GmRhoGAP4*) and in root and stem (for *GmRhoGAP16*) compared to other tissues (Figure 6 and Table S8). Furthermore, *GmRhoGAP* genes generally exhibited lower expression levels in pods and flowers (Figure 6 and Table S8). These findings suggest that *GmRhoGAP* genes are highly active in most soybean tissues and may be broadly involved in regulating various physiological processes.

### 2.7. Response of GmRhoGAPs to Rhizobial Infection

**Figure 7:**
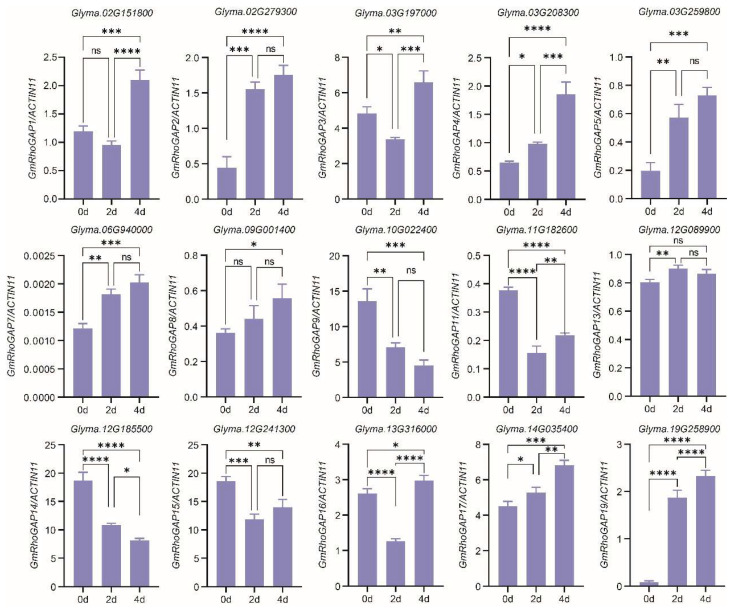
The expression levels of *GmRhoGAPs* at 0 d, 2 d, and 4 d after inoculation with rhizobia. *, **, ***, and **** indicate significant differences at the levels of *p* < 0.05, *p* < 0.01, *p* < 0.001, and *p* < 0.0001, respectively. ns means not significant.

To investigate whether *GmRhoGAP* genes exhibit transcriptional changes in response to *Bradyrhizobium japonicum* USDA110 infection, soybean plants were inoculated with the bacteria, and root samples were collected at 0, 2, and 4 days post-inoculation (dpi) for RNA extraction. The expression levels of 15 *GmRhoGAP* genes were measured by qRT-PCR, and the data at 0 dpi were compared with those at 2 and 4 dpi. The results showed that, with the exception of *GmRhoGAP13*, the expression levels of the other 14 *GmRhoGAP* genes changed significantly after inoculation. Among them, 10 *GmRhoGAP* genes were upregulated after inoculation. Specifically, *GmRhoGAP1* was downregulated at 2 dpi compared to 0 dpi, though not significantly, but was significantly upregulated at 4 dpi. *GmRhoGAP2*, *GmRhoGAP5*, and *GmRhoGAP7* rapidly responded at 2 dpi and then stabilized. *GmRhoGAP4*, *GmRhoGAP17* and *GmRhoGAP19* remained continuously activated, with significant upregulation throughout the infection process. *GmRhoGAP3* and *GmRhoGAP16* were briefly suppressed at 2 dpi but reactivated at 4 dpi. *GmRhoGAP8* showed significantly higher expression levels after inoculation, though it remained at a low and stable level during the infection period (Figure 7). In contrast, 4 genes exhibited a downregulation in overall expression after inoculation. *GmRhoGAP14* continuously decreased. *GmRhoGAP11* was significantly downregulated at 2 dpi, with a recovery at 4 dpi, though its level was still lower than the control level. *GmRhoGAP9* and *GmRhoGAP15* were suppressed at 2 dpi, with significant downregulation, and maintained stable levels at 4 dpi (Figure 7). These findings suggest that *GmRhoGAP* genes display highly differentiated expression patterns during rhizobial infection, which is potentially linked to distinct physiological stages of symbiosis establishment.

## 3. Discussion

RhoGAPs function as negative regulators of Rho GTPases, stimulating the intrinsic GTPase activity of Rho GTPases, thereby promoting GTP hydrolysis [22]. Plant Rho GTPases were first cloned and identified from *Pisum sativum* in 1993 [23]. Due to the close relationship between plant Rho proteins and the Rac subfamily of animal Rho proteins, they were later named Rac or Rop [24,25]. To date, more than 70 RhoGAPs have been identified in eukaryotes, including 66 RhoGAP proteins in humans [26,27]. In contrast, the *RhoGAP* family in plants is smaller, and research began relatively later. The first cloning and identification of *AtRopGAP1* and its homologs in *A. thaliana* occurred in 2000 [12]. With the advancement of genomics, the identification of plant *RhoGAP* genes has gradually progressed. Recently, 19 *RhoGAP* genes were identified in *O. sativa* [28]. However, research has mainly focused on model plants, and the comprehensive identification of *RhoGAPs* in *G. max* as well as their functions during nodulation remain unclear. Therefore, a thorough identification and functional analysis of the *GmRhoGAP* gene family is crucial for understanding their role in symbiotic nitrogen fixation.

In this study, phylogenetic and synteny analyses revealed that the GmRhoGAP family exhibits both interspecies conservation and distinct lineage-specific divergence. The phylogenetic framework was constructed using representative species from monocots (*O. sativa*), dicots (*A. thaliana*), and legumes (*G. max*) to provide a broad evolutionary perspective across major angiosperm lineages. Phylogenetic analysis of RhoGAPs from *A. thaliana*, *O. sativa*, and *G. max* showed that the RhoGAP proteins in these analyzed species can be classified into four major clades; however, these clades exhibited marked differences in species composition (Figure 1). Clades I and II only contain rice RhoGAPs, suggesting these clades may have undergone species-specific differentiation [29]; meanwhile, Clades III and IV include RhoGAPs from *G. max*, *A. thaliana*, and *O. sativa*, indicating that these genes likely formed and remained relatively conserved before the divergence of monocots and dicots (Figure 1) [30]. This sampling strategy provides a foundational framework for macroevolutionary analyses of the *RhoGAP* gene family in angiosperms, which facilitates the interpretation of its major evolutionary trends. Future studies can further incorporate additional legume genome data to improve the resolution of intra-legume evolutionary analyses of the *RhoGAP* gene family and to further refine our understanding of its evolutionary patterns within legumes.

Synteny analysis revealed 20 pairs of segmentally duplicated genes within the soybean genome, with no tandem duplicated genes identified, suggesting that segmental duplication is the main mechanism for the expansion of the *GmRhoGAP* family (Figure 4A and Table S5) [31]. Additionally, *GmRhoGAPs* share pairwise synteny with genes in *A. thaliana* and *O. sativa*, indicating these genes underwent specific evolutionary events after the divergence of the three species (Figure 4B and Table S6) [29].

Significant differences in the physicochemical properties of GmRhoGAP proteins were observed. The protein lengths of GmRhoGAPs range from 151 to 946 amino acids (Table S4), similar to the lengths of RhoGAP proteins in *O. sativa* [28]. Moreover, GmRhoGAPs include 10 conserved motifs and have typical exon-intron structures (Figure 2A,C and Figure S1). Based on domain composition, GmRhoGAPs can be classified into three types: Type I with both CRIB and GAP domains; Type II with PH and GAP domains; and Type III with only the GAP domain (Figure 2B). This domain composition pattern is also present in *Capsicum* [32]. Further integration of analyses on motif composition, conserved domains, and gene structure revealed that GmRhoGAPs within the same phylogenetic branch exhibit high consistency in motif arrangement, domain composition, exon number, and protein length, whereas notable differences exist between branches. These structural differences may drive functional divergence among family members (Figure 2).

*Cis*-acting regulatory elements play key roles in plant growth, development, and environmental responses, participating in processes such as hormone signaling, stress responses, and developmental regulation [33]. Previous studies have shown that anaerobic induction elements regulate gene expression related to flooding stress, helping rice adapt to hypoxic environments [34]; MeJA-responsive elements regulate gene expression related to disease resistance, enhancing *Arabidopsis* disease defense [35]; and light-responsive elements regulate plant responses to light, influencing photosynthesis and growth regulation [33]. In this study, 15 *cis*-regulatory elements were identified in the promoter regions of *GmRhoGAPs*, with high frequencies of anaerobic induction elements, MeJA-responsive elements, and light-responsive elements, suggesting that *GmRhoGAPs* may be involved in processes such as hypoxia response, disease defense, and light signaling regulation in soybean (Figure 5 and Table S7).

As a globally important food and oilseed crop, soybean establishes a symbiotic nitrogen fixation system with rhizobia, which plays a crucial role in agricultural production. This study found that the expression levels of most *GmRhoGAPs* significantly changed after inoculation with rhizobia. Some genes, such as *GmRhoGAP4*, *GmRhoGAP8*, *GmRhoGAP17*, and *GmRhoGAP19* were consistently upregulated throughout the inoculation process, indicating that they may act as positive regulators and potentially participate in the establishment and maintenance of symbiosis. Meanwhile, the majority of *GmRhoGAP* genes exhibited rapid responses at 2 dpi, followed by stabilization or stage-specific expression changes, indicating their potential involvement in early signal perception or regulation of the infection process (Figure 7). In addition, *GmRhoGAP9*, *GmRhoGAP11*, *GmRhoGAP14*, and *GmRhoGAP15* were significantly downregulated after infection, implying that they may function as negative regulators in symbiotic signaling, possibly by attenuating Rop GTPase activity during specific stages of infection (Figure 7). In conclusion, *GmRhoGAPs* exhibit pronounced temporal dynamics and functional divergence during rhizobial infection, indicating that they may fine-tune the Rop signaling pathway through both positive and negative regulatory roles, thereby contributing to the spatiotemporal control of cellular processes during symbiosis establishment and nodulation. However, further functional validation is required to clarify their precise roles in nodule development and symbiotic nitrogen fixation.

## 4. Materials and Methods

### 4.1. Plant Materials and Growth Conditions

The soybean cultivar ‘Williams 82’ was grown in a greenhouse at 25 °C, 70% relative humidity, and a photoperiod of 16 h light/8 h dark. Surface-cleaned seeds were germinated in vermiculite. Seven-day-old seedlings, at the stage when the trifoliate leaves were fully expanded, were inoculated with 30 mL of *Bradyrhizobium japonicum* USDA110 suspension at an optical density of OD_600_ = 0.8 per plant. Root samples were collected at 0, 2, and 4 dpi. The samples were immediately wrapped in aluminum foil, frozen in liquid nitrogen, and stored at −80 °C until further use. Total RNA was extracted from root tissues, and three independent biological replicates were included for each time point.

### 4.2. Identification of GmRhoGAPs

To identify RhoGAP protein sequences in the *G. max* genome (Wm82.a2.v1), the BLASTP algorithm in the Phytozome 14 database (https://phytozome-next.jgi.doe.gov/ accessed on 3 February 2026) was used with the rice RhoGAP1 protein sequence as the query [28]. Stringent screening criteria were applied, including a sequence identity ≥50% and an E-value ≤ 1.0 × 10^−40^. To further expand the candidate dataset, the conserved domain of OsRhoGAP1 was identified from the InterPro database (https://www.ebi.ac.uk/interpro/ accessed on 3 February 2026), corresponding to the Pfam entry PF00620. The Hidden Markov Model (HMM) profile was then used to search the soybean proteome in TBtools (v2.472) [36]. All candidate protein sequences obtained from the two approaches were subsequently subjected to conserved domain validation using SMART (https://smart.embl.de/ accessed on 4 February 2026), CDD (https://www.ncbi.nlm.nih.gov/cdd/ accessed on 4 February 2026), and UniProt (https://www.uniprot.org/ accessed on 4 February 2026). The detailed information of all identified genes is provided in Table S1.

### 4.3. Physicochemical Property Analysis of GmRhoGAP Proteins

To characterize the physicochemical properties of GmRhoGAP proteins, including aa, MW, pI, II, AI and GRAVY, all protein sequences were analyzed using the ProtParam tool available on the ExPASy server (https://web.expasy.org/protparam/ accessed on 5 February 2026).

### 4.4. Phylogenetic Analysis of GmRhoGAPs

RhoGAP protein sequences from *A. thaliana* and *O. sativa* were retrieved from the Phytozome 14 database. Multiple sequence alignment was performed using the ClustalW algorithm [37]. A phylogenetic tree was subsequently constructed based on the full-length protein sequences of AtRhoGAPs, OsRhoGAPs, and GmRhoGAPs using the Neighbor-Joining (NJ) method implemented in MEGA11, with 1000 bootstrap replicates. The resulting tree was visualized and annotated using Evolview (https://www.evolgenius.info/evolview/#/treeview accessed on 7 February 2026).

### 4.5. Gene Structure, Conserved Motif, and Domain Analysis of GmRhoGAPs

The gene structure of 19 *GmRhoGAP* genes was analyzed based on the soybean genome annotation (GFF3 file) and visualized using TBtools (v2.472) [36]. Conserved motifs were predicted using the MEME suite (https://meme-suite.org/meme/tools/meme accessed on 11 February 2026), with the maximum number of motifs set to 10 and all other parameters set to default values. Conserved domains were identified using the CDD tool. The results from MEME and CDD analyses were subsequently visualized using TBtools (v2.472) [36].

### 4.6. Chromosomal Distribution and Synteny Analysis

To determine the chromosomal locations of the 19 *GmRhoGAP* genes, their physical positions were visualized using Gene Location Visualize from the GTF/GFF function in TBtools (v2.472) [36]. Gene duplication events within the *GmRhoGAP* family were identified using the One-Step MCScanX function implemented in TBtools (v2.472) [36]. The nonsynonymous substitution rate (Ka), synonymous substitution rate (Ks), and their ratio (Ka/Ks) for collinear gene pairs were calculated using the Simple Ka/Ks Calculator in TBtools (v2.472) [36]. The divergence time (T) was estimated using the formula T = Ks/(2 λ), where the synonymous substitution rate (λ) for soybean was set to 6.161029 [38]. Comparative synteny analysis between soybean and other species was performed using the Multiple Synteny Plot function in TBtools (v2.472) [36].

### 4.7. Cis-Acting Regulatory Element Analysis

The 2000 bp upstream genomic sequences of *GmRhoGAP* genes were retrieved from the soybean genome database and analyzed using the PlantCARE online tool (http://bioinformatics.psb.ugent.be/webtools/plantcare/html/ accessed on 13 February 2026) to identify putative *cis*-acting regulatory elements. The identified *cis*-elements were subsequently visualized using TBtools (v2.472) [36].

### 4.8. Expression Analysis of GmRhoGAPs in Different Tissues

To investigate the expression patterns of *GmRhoGAP* genes across different tissues, Fragments Per Kilobase of transcript per Million mapped reads (FPKM) values were obtained from the Phytozome 14 database. Expression profiles were visualized using the HeatMap function in TBtools (v2.472) [36].

### 4.9. Expression Analysis of GmRhoGAP Genes Under Rhizobial Infection

Total RNA was extracted using the FastPure Universal Plant Total RNA Isolation Kit (Vazyme Biotech Co., Ltd., Nanjing, China) (RC411-01). First-strand cDNA was synthesized using the HiScript III qRT SuperMix Kit (Vazyme Biotech Co., Ltd., Nanjing, China) (R323-01), followed by tenfold dilution and storage at −80 °C. Gene-specific primers were designed for 15 *GmRhoGAP* genes, and their specificity was verified by agarose gel electrophoresis of qRT-PCR products. The qRT-PCR was performed using the ChamQ SYBR qPCR Master Mix (Vazyme Biotech Co., Ltd., Nanjing, China) (Q711-02). Each qRT-PCR reaction was carried out in a total volume of 10 μL, which contained 5 μL of 2 × ChamQ Universal SYBR qPCR Master Mix, 2.4 μL of deionized water, 2 μL of diluted cDNA, and 0.3 μL of each forward and reverse primer (10 μmol·L^−1^). The qPCR amplification program was as follows: initial denaturation at 95 °C for 1 min; followed by 40 cycles of 95 °C for 5 s and 60 °C for 30 s; and a melt curve stage of 95 °C for 15 s, 60 °C for 1 min, and 95 °C for 15 s. *GmACTIN11* was used as the internal reference gene, and relative gene expression levels were calculated using the 2^−ΔΔCt^ method [39]. Three biological replicates were included for each treatment. Primer sequences are listed in Table S2. Statistical analysis of gene expression data was performed using GraphPad Prism 9.5. One-way analysis of variance (one-way ANOVA) was used to evaluate differences among groups, followed by multiple comparison tests.

## 5. Conclusions

In this study, a total of 19 *GmRhoGAP* genes were identified and systematically characterized. These genes exhibit diverse motif compositions and gene structural features. All *GmRhoGAPs* were distributed across 11 chromosomes in the soybean genome. Intra-genomic synteny analysis identified 20 pairs of *GmRhoGAP* genes, all of which originated from segmental duplication events. *Cis*-regulatory element analysis of promoter regions suggests that *GmRhoGAPs* may participate in cell development, phytohormone signaling, environmental stress responses, and light-responsive processes. Tissue-specific expression profiling and qRT-PCR analysis revealed that *GmRhoGAPs* display distinct expression patterns across different tissues and exhibit differential responses at various stages of rhizobial infection. Overall, this study provides new insights into the evolutionary patterns and functional diversification of the *GmRhoGAP* gene family, and lays a foundation for future investigations into their roles in nodule development. These findings also highlight the potential application value of *GmRhoGAPs* in the genetic improvement of legume crops.

## Data Availability

All data analyzed during this study are included in this published article and its Supplementary Information Files.

## Supplementary Materials

The following supporting information can be downloaded at: https://www.mdpi.com/article/10.3390/ijms27146239/s1.
