# Supplementary material for: Identification of RhoGAP Gene Family in Soybean (Glycine max L.) and Its Role in the Response to Rhizobium Infection
Source: Int J Mol Sci. 2026 Jul 13;27(14):6239. doi: 10.3390/ijms27146239 (PMC13410095; doi:10.3390/ijms27146239)
Supplement: Supplementary file 1 [file ijms-27-06239-s001.zip › Supplementary Figure.pdf]

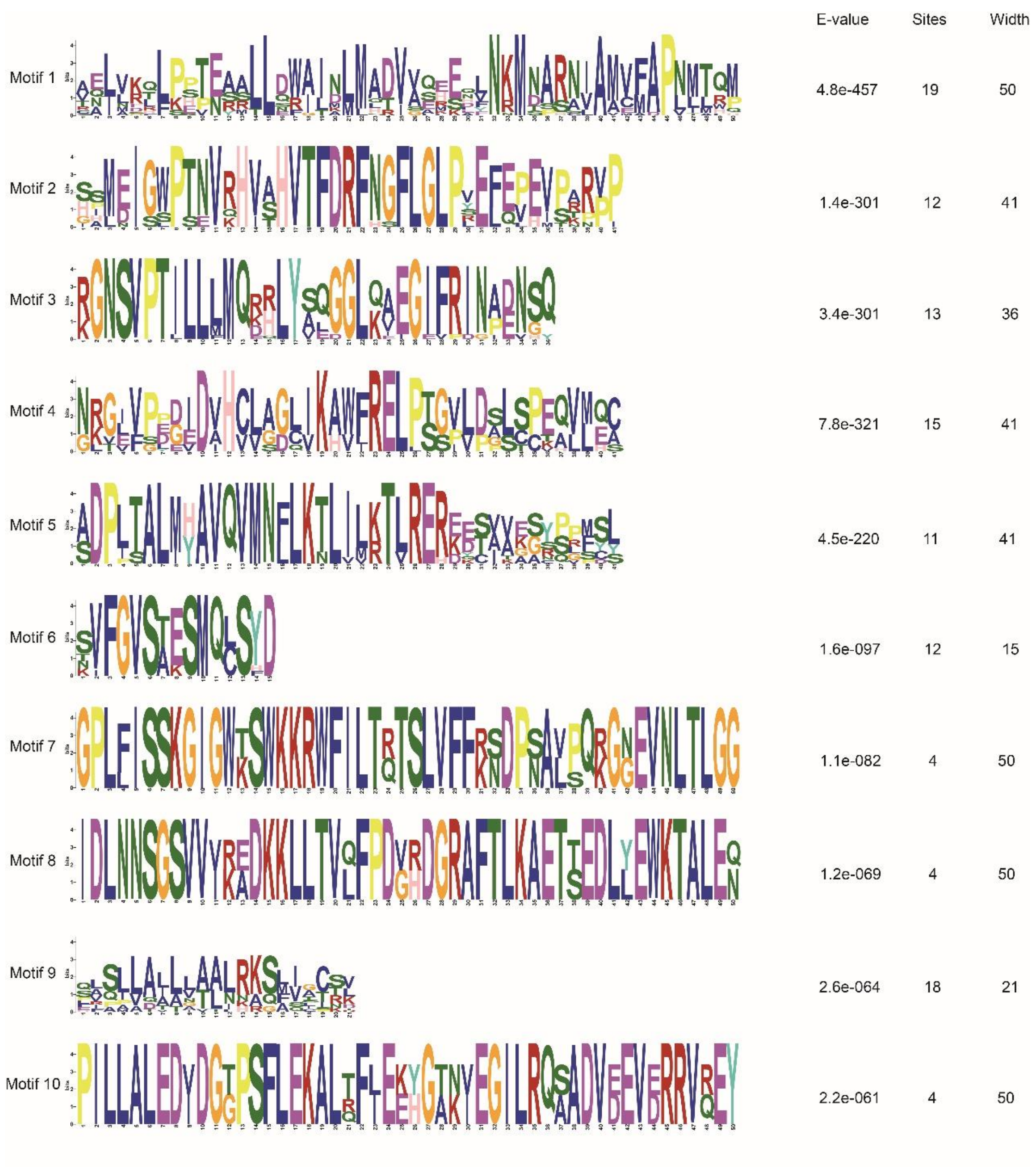

Supplementary Figure S1. The information of identified 10 motifs in GmRhoGAP proteins.
